# Predicting diabetic kidney disease for type 2 diabetes mellitus by machine learning in the real world: a multicenter retrospective study

**DOI:** 10.3389/fendo.2023.1184190

**Published:** 2023-07-04

**Authors:** Xiao zhu Liu, Minjie Duan, Hao dong Huang, Yang Zhang, Tian yu Xiang, Wu ceng Niu, Bei Zhou, Hao lin Wang, Ting ting Zhang

**Affiliations:** ^1^ Department of Cardiology, the Second Affiliated Hospital of Chongqing Medical University, Chongqing, China; ^2^ Medical Data Science Academy, Chongqing Medical University, Chongqing, China; ^3^ College of Medical Informatics, Chongqing Medical University, Chongqing, China; ^4^ Information Center, The University-Town Hospital of Chongqing Medical University, Chongqing, China; ^5^ Department of Nuclear Medicine, Handan First Hospital, Hebei, China; ^6^ Department of Endocrinology, Fifth Medical Center of Chinese People's Liberation Army (PLA) Hospital, Beijing, China

**Keywords:** type 2 diabetes mellitus, diabetic kidney disease, machine learning, prediction, CatBoost model

## Abstract

**Objective:**

Diabetic kidney disease (DKD) has been reported as a main microvascular complication of diabetes mellitus. Although renal biopsy is capable of distinguishing DKD from Non Diabetic kidney disease(NDKD), no gold standard has been validated to assess the development of DKD.This study aimed to build an auxiliary diagnosis model for type 2 Diabetic kidney disease (T2DKD) based on machine learning algorithms.

**Methods:**

Clinical data on 3624 individuals with type 2 diabetes (T2DM) was gathered from January 1, 2019 to December 31, 2019 using a multi-center retrospective database. The data fell into a training set and a validation set at random at a ratio of 8:2. To identify critical clinical variables, the absolute shrinkage and selection operator with the lowest number was employed. Fifteen machine learning models were built to support the diagnosis of T2DKD, and the optimal model was selected in accordance with the area under the receiver operating characteristic curve (AUC) and accuracy. The model was improved with the use of Bayesian Optimization methods. The Shapley Additive explanations (SHAP) approach was used to illustrate prediction findings.

**Results:**

DKD was diagnosed in 1856 (51.2 percent) of the 3624 individuals within the final cohort. As revealed by the SHAP findings, the Categorical Boosting (CatBoost) model achieved the optimal performance 1in the prediction of the risk of T2DKD, with an AUC of 0.86 based on the top 38 characteristics. The SHAP findings suggested that a simplified CatBoost model with an AUC of 0.84 was built in accordance with the top 12 characteristics. The more basic model features consisted of systolic blood pressure (SBP), creatinine (CREA), length of stay (LOS), thrombin time (TT), Age, prothrombin time (PT), platelet large cell ratio (P-LCR), albumin (ALB), glucose (GLU), fibrinogen (FIB-C), red blood cell distribution width-standard deviation (RDW-SD), as well as hemoglobin A1C(HbA1C).

**Conclusion:**

A machine learning-based model for the prediction of the risk of developing T2DKD was built, and its effectiveness was verified. The CatBoost model can contribute to the diagnosis of T2DKD. Clinicians could gain more insights into the outcomes if the ML model is made interpretable.

## Introduction

Diabetes mellitus refers to a chronic epidemic metabolic disease with high blood glucose. The latest statistics from the International Diabetes Federation (IDF) suggested that approximately 463 million adults (aged 20-79 years) worldwide would have diabetes by 2019; the number of people with diabetes was estimated to reach 700 million by 2045 (1). Complications of diabetes have been found as the leading cause of death in diabetic patients (2), with 76.4% of diabetic patients reporting at least one complication (3). Diabetic kidney disease (DKD) has been reported as a main microvascular complication of diabetes mellitus, which is characterized by high prevalence, mortality, and treatment costs, but low awareness and poor prevention and treatment rates (4). In China, nearly 20-40% of diabetic patients suffer from DKD, while the awareness rate of DKD is lower than 20%, and the treatment rate is even lower than 50% (5).

The typical progression of DKD refers to an initial increase in urinary albumin excretion (called microalbuminuria), which is accompanied with progression to massive albuminuria and subsequent rapid decline in renal function. As a result, proteinuria has been considered the initial pathway for the progression of declining renal function from the traditional perspective (6). However, the above theory has been challenged since numerous patients with proteinuria have been found to return to normal albumin excretion rates either spontaneously or based on the integrated risk management with DKD (7–11). On that basis, the effectiveness of microalbuminuria as a traditional marker of DKD and the optimal opportunity for intervention are challenged since DKD is generally insidious during onset (12). Although renal biopsy is capable of distinguishing DKD from Diabetic kidney disease (NDKD), no gold standard has been validated to assess the development of DKD. Although increased screening frequency can avoid delayed diagnoses, this is not uniformly implemented. Furthermore, the prevention, early diagnosis and treatment of DKD take on a critical significance in reducing the incidence of cardiovascular events in diabetic patients and improving their survival and quality of life. Accordingly, there is an urgent need for a simple and convenient clinical tool to assess DKD in daily clinical practice.

Developing a risk scoring system based on simple predictors, i.e., clinical data, is considered a vital for monitoring and diagnosing DKD. Machine learning algorithm (ML) show significant advantages in processing a considerable number of data with high-dimensional properties and numerous cases. It is extensively employed for disease prediction (13). Machine learning algorithms can efficiently predict the DKD (14–17). Identification of risk factors for the progression of DKD to ESRD is expected to improve the prognosis by early detection and appropriate intervention (18). Most studies on predictive models for DKD have adopted a single classifier for statistical analysis, and most of them achieved small sample sizes. Under excessive samples and variables, the models will be prone to underfitting or overfitting, and the performance and efficiency could be enhanced. Most of the prediction models developed by foreign researchers apply to the white population, and they are likely to be less applicable to the Asian population (19, 20). Thus, it is of clinical significance in developing ancillary diagnostic models for DKD with the use of ML. However, few large-scale studies have investigated the use of machine learning analysis of clinical characteristics to predict DKD in the Chinese population. A retrospective cohort study was conducted, which involved the collection of clinical parameters and the application of machine learning models to differentiate between DKD and NDKD.

## Materials and methods

### Study population

The data originated from Chongqing Medical University’s Medical Data Intelligence Platform(Yidu-Cloud (Beijing) Technology Co, Ltd, China), which consisted of the data from seven institutions and over 40 million electronic medical records (during 1 January 2010 to 31 May 2020) (21–23). Only the information from the first hospitalization was applied in the event of subsequent hospitalizations. Xiaozhu Liu (Account Number: cy2014223346)and Minjie Duan (Account Number: MI2020111943) were permitted to access data directly on the platform system where all information is anonymous and has a unique identifying code to preserve privacy, while an informed consent from the patient is unnecessary. The local institutional ethics committee gave us their authorization.The inclusion and exclusion criteria below were employed for screening:

Inclusion criteria: (1) hospitalization for T2DM or T2DKD; (2) following the WHO 1999 diagnostic criteria for diabetes mellitus; (3) age >18 years; following the diagnostic criteria of the KDOQI US commentary on the 2012 KDIGO clinical practice guideline for Chronic kidney disease (24).

Exclusion criteria: (1) combination of other possible complications such as urinary tract infection, malignancy; (2) immune diseases (e.g., systemic lupus erythematosus and vasculitis); (3) Other endocrine diseases; (4) type 1 diabetes, gestational diabetes and other diabetes with unclear classification; (5) hospitalization days <1; (6) discharge against medical advice; (7) patients lost to follow-up or death during index hospitalization; and (8) patients with >25% of data missing.

**Figure 1:**
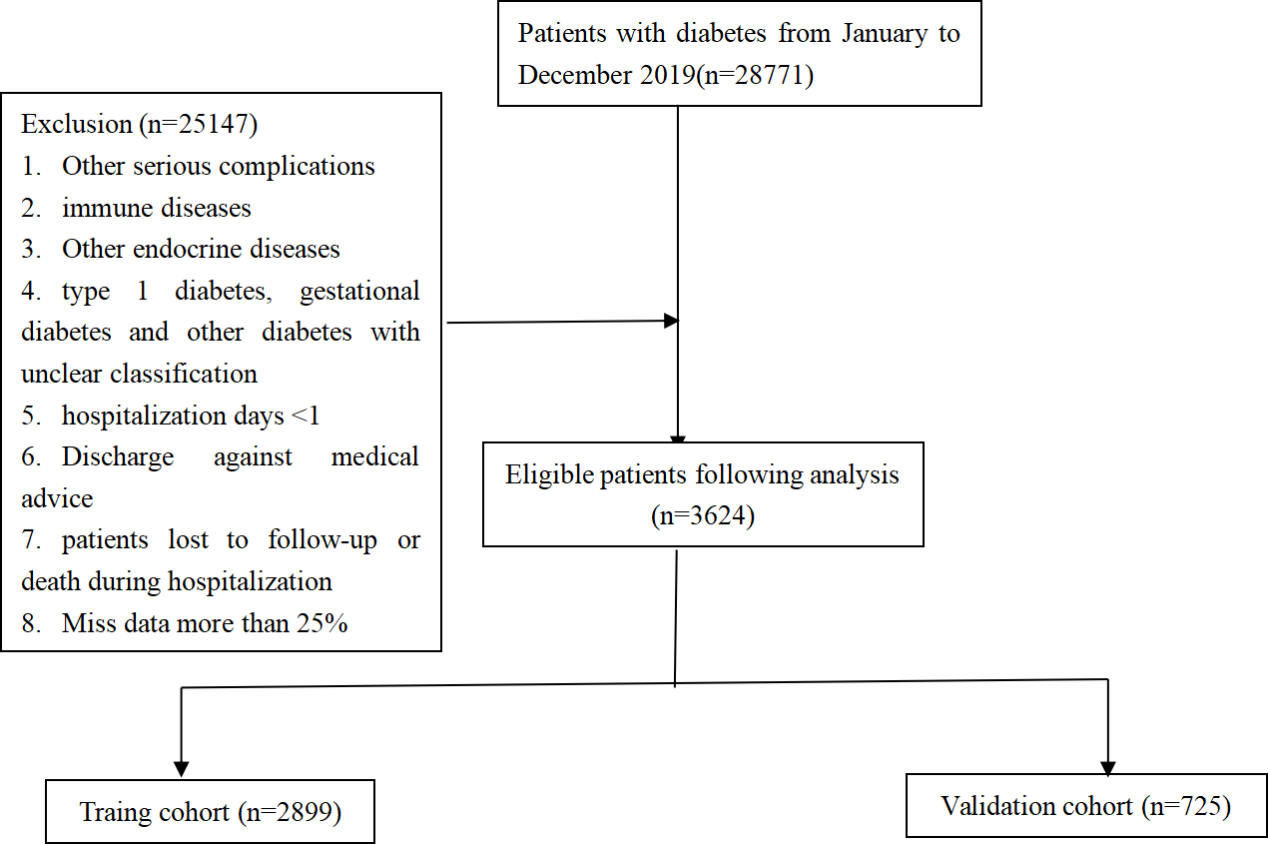
The flowchart of the study.

In accordance with the inclusion and exclusion criteria, 3624 patients with T2DM were recruited, which consisted of 1856 patients with T2DKD (**Figure 1**). In this study, DKD was defined in accordance with the National Health Insurance Administration’s definition of catastrophic illness ICD-9 and ICD-10 codes for DKD.

The definition of CKD in the 2012 KDIGO clinical practice guideline was adopted (24, 25).

### Data collection and data preprocessing

The latest literature on DKD was reviewed and combined with clinical experience to acquire relevant clinical data and laboratory characteristics (25–28). 53 clinical characteristics with missing values ≤ 25% were covered. Since most models cannot analyze data with missing values, Multivariate Imputation by Chained Equations (MICE) algorithm was used for data filling.

Baseline data were compared in patients with DKD and T2DKD from the first examination and test results after admission (**Table S1**)

### Model development and performance evaluation

The data set was randomly assigned to a training set (80%) and a validation set (20%) based on stratified random sampling. Our models were developed using the training set, and their performance was assessed using the validation set. The least absolute shrinkage and selection operator (LASSO) was employed for selecting the risk predictors to eliminate unnecessary and redundant information and increase the model’s discriminative capacity. Finally, non-zero regression coefficient variables were selected to build the prediction models.

To select the prospective algorithms for our prediction models, we first analyzed the performance of 15 machine learning algorithms without hyper-parameters tuning. After that, an algorithm with the optimal performance was selected in accordance with the model’s accuracy and the area under the receiver operating characteristic curve (AUC). PyCaret (version 2.3.3), an open source, low-code machine learning library in Python, was employed to perform the screening procedure. Second, the Bayesian Optimization approach with 10-fold cross validation was adopted for adjusting a prediction model based on the training set to find the ideal hyper-parameter configuration. The above algorithm is an efficient constrained global optimization tool, which was performed based on the functions of the bayes_opt Python package (version 1.2.0). AUC, accuracy and sensitivity were obtained to assess the models performance based on the independent validation set.

To reduce the black-box nature of machine learning and to allow clinicians to understand the results of the provided model, SHapley Additive exPlanations (SHAP) was adopted to interpret the model with the use of SHAP python package (version 0.39.0). The significance of input features was obtained with the use of a game-theoretic algorithm based on the independent validation set. It is noteworthy that all 38 variables would not always be available in clinical practice. Accordingly, the top 12 were taken from SHAP summary plots to build the simpler model, and the discriminative power was compared between the full model and simpler models.

### Statistical analysis

For baseline comparison of data sets, categorical variables were denoted as percentages, and Chi-square test or Fisher’s exact test was performed for comparison between groups. Continuous variables were examined for normality using Kolmogorov-Smirnov test, and measures following normal distribution were denoted as mean ± standard deviation, and Student’s t-test was used for comparison between groups, and measures not following normal distribution were denoted as median (interquartile range), and Mann-Whitney U rank sum test was performed for comparison between groups. R (version 4.0.2) was adopted for statistical analysis. A two-sided P < 0.05 was considered to achieve statistical significance.

## Results

### Patient characteristics

The data were assigned to a training set and a validation set at 8:2. The training set consisted of 2899 cases, including 1485 cases of T2DKD (51.2%) and 1414 cases of T2DM (48.8%); the validation set consisted of 725 cases, including 371 cases of T2DKD (51.2%) and 354 cases of T2DM (48.8%) (see **Table S2** for details).

### Feature selection

**Figure 2:**
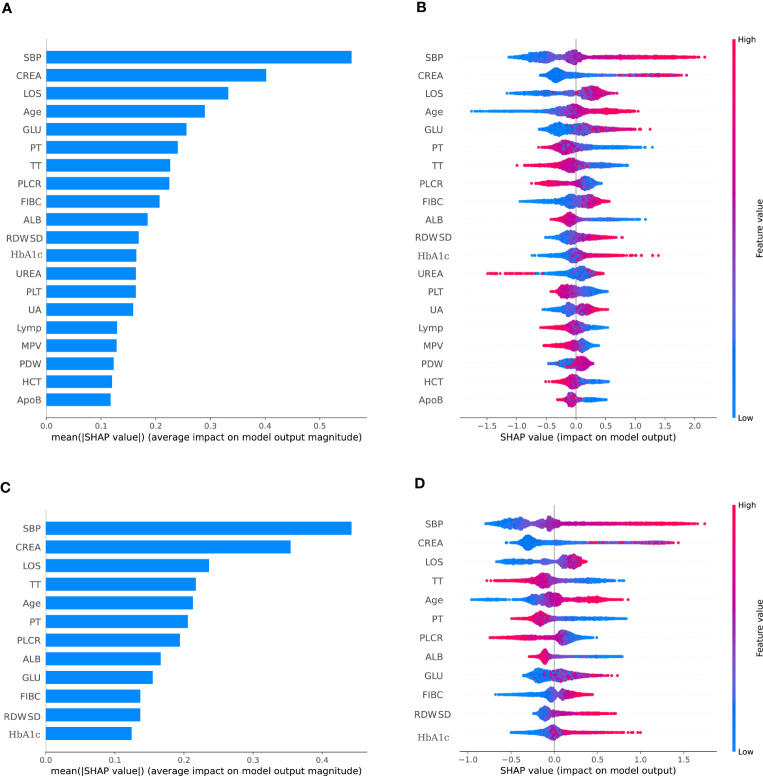
The SHAP summary plots for the CatBoost model. **(A)** depicts the most 38 effective characteristics on prediction (ranked from showing the highest to lowest importance). **(B)** depicts the distribution of the effects of 38 characteristics on the output of the model. **(C)** depicts the most 12 effective characteristics on prediction (ranked from showing the highest to lowest importance). **(D)** depicts the distribution of the effects of 12 characteristics on the output of the model.For numeric characteristics, the colors indicate the feature values: red for larger values and blue for smaller values. The thickness of the line is defined by the number of instances at a specific value, and it is made up of individual dots representing each DKD (e.g., most patients have a low risk of developing of DKD). A lower likelihood is indicated by a negative SHAP value (stretching to the left), while a higher probability is indicated by a positive SHAP value (reaching to the right). The gray dots reflect particular possible values for non-numeric characteristics such as main diagnosis, with select diagnoses considerably raising or decreasing the model’s output, while the majority of diagnoses have just a little influence on prediction.

Least absolute shrinkage and selection operator(LASSO) was employed to select the most significant features, so as to classify individuals diagnosed DKD. All features (a total of 53 variables) were included in the LASSO regression analysis and narrowed down to 38 features with non-zero β coefficients in the LASSO regression model. The above features were Sex, Smoke, Drink history, Age, length of stay (LOS), Systolic blood pressure (SBP), diastolic blood pressure (DBP), total protein (TP), albumin (ALB), gamma glutamyltransferase (GGT), alanine aminotransferase (ALT), alkaline phosphatase (ALP), total cholesterol (TC), triglyceride (TG), high-density lipoprotein cholesterol (HDL-C), phosphorus (P), glucose (GLU), apolipoprotein A1 (ApoA1), Hemoglobin A1C (HbA1C), creatinine (CREA), urea, uric acid (UA), fibrinogen (FIB-C), platelet count (PLT), prothrombin time (PT), thrombin time (TT), monocyte percentage (Mon%), basophil count (Bas), eosinophil count (Eos), neutrophil count (Neu), platelet large cell ratio (P-LCR), mean corpuscular volume (MCV), mean corpuscular hemoglobin concentration (MCHC), lymphocyte count (Lym), red blood cell distribution width-standard deviation (RDW-SD), hematocrit (HCT), platelet distribution width (PDW), mean platelet volume (MPV) (**Figure 2A**).

### Performance of models in predicting DKD

**Figure 3:**
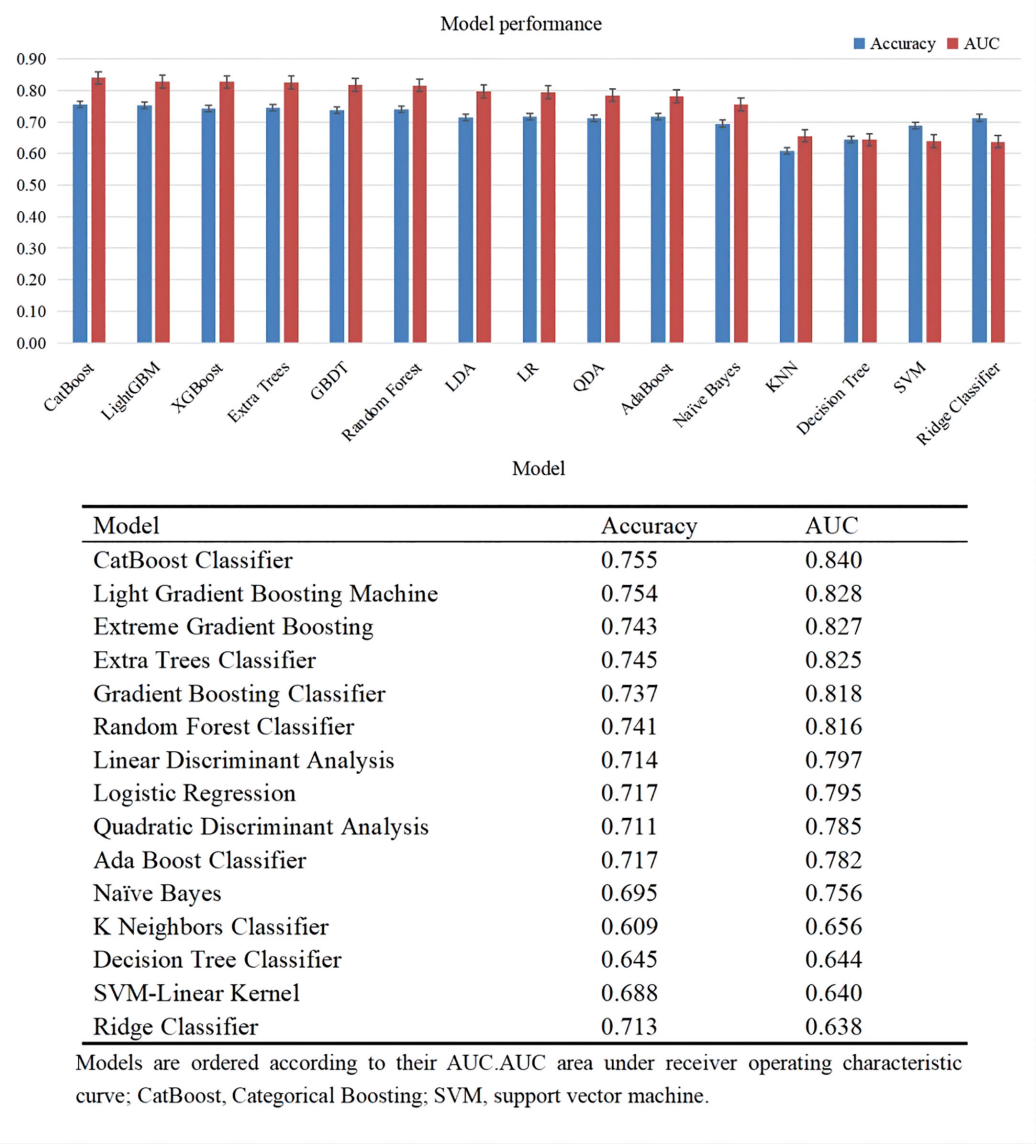
Performance of different models in internal validation.

**Figure 3** lists the predictive performance of 15 ML models after 10-fold cross validation for internal training. Almost all classic ML methods capable of conducting classification analysis were considered. The top six models consisted of CatBoost Classifier, Light Gradient Boosting Machine, extreme gradient Boosting, Extra Trees Classifier, Gradient Boosting Classifier, Random Forest Classifier, with AUC over 0.8. As revealed by the results, the CatBoost model indicated the maximum performance in predicting DKD risk with AUC and accuracy of 0.840 and 0.755, respectively. As a result, the CatBoost model was selected and optimized in the following step.

**Figure 4:**
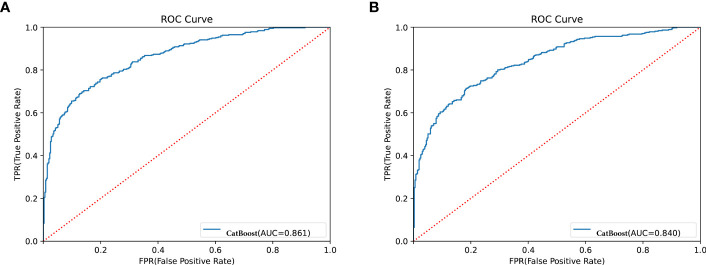
Receiver operator characteristic (ROC) curves for the CatBoost model. **(A)** Shows ROC for CatBoost with most 38 effective characteristics on prediction (ranked from most to least important). **(B)** Shows ROC for CatBoost with most 12 effective characteristics on prediction (ranked from most to least important).

Bayesian optimization algorithm with 10-fold cross validation to select the optimal hyperparameter configuration for the CatBoost model. The optimized CatBoost model exhibited the optimal and the most stable performance, with an AUC of 0.861, an accuracy of 0.777, a sensitivity of 0.755 (**Figure 4**). To increase the transparency and usability in real clinical setting of the prediction model, 12 top features were selected to construct the simpler prediction model based on the SHAP values and clinical availability. The top 12 most significant features consisted of SBP, CREA, LOS, TT, Age, PT, PLCR, ALB, GLU, FIBC, RDWSD, HbA1c (**Figure 2C**). As depicted in **Figure 4**, the simpler CatBoost model showed slight worse performance (AUC: 0.840). In this study, our CatBoost model was illustrated using the SHAP method by Lundberg and Lee (29). We employ the Shap technique to gain a global interpretation of our reserved cohort as well as individual patient interpretations. The SHAP summary plots for the top 38 clinical characteristics contributing to our ML model’s prediction of DKD development in our research are shown in **Figures 2A, B**. The SHAP summary plots for the top 12 clinical characteristics contributing to our ML model’s prediction of developing DKD in our research are shown in **Figures 2C, D**.

**Figure 5:**
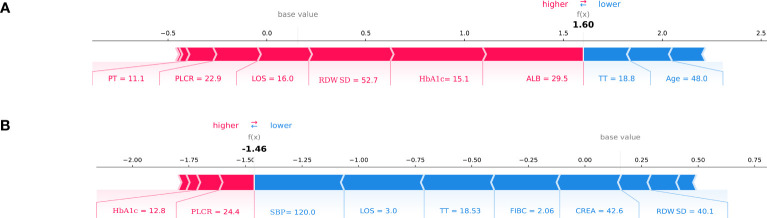
SHAP force plot for two patients of the held-out validation set. **(A)** patient at high risk of developing T2DKD; **(B)** patient at low risk of developing T2DKD. DKD, diabetic kidney disease; ALB, albumin; HbA1C, hemoglobin A1C; RDWSD, red blood cell distribution width-standard deviation; LOS, length of stay; PLCR, platelet large cell ratio; PT, prothrombin time; TT, thrombin time; SBP, Systolic blood pressure; FIBC,fibrinogen; CREA, creatine.

Meanwhile, we show the SHAP explanation force diagram for two patients from the CatBoost model’s validation set (**Figure 5**). **Figure 5A** depicts a patient who is 48 years old. This patient’s anticipated risk of having DKD is significant, at 160 percent, in comparison with a baseline risk of 10%. (average prevalence of the validation cohort). Lower ALB of 29.5g/l, increased HbA1C of 15.1 percent, increased RDWSD of 52.7 mg/dl, prolonged LOS of 16 days, lower PLCR of 22.9 percent, and PT of 11.1 seconds were the characteristics found by the model for the prediction of a greater prevalence in this patient. The patient’s age of 48 years and TT of 18.8 seconds help to mitigate the increased risk. **Figure 5B** presents another T2DM patient. This patient’s anticipated risk of getting DKD was -146 percent, in comparison with a baseline risk of 10%. (average prevalence of the validation cohort). Normal SBP of 120mmHg, shorter LOS of 3 days, normal TT of 18.53 seconds, normal FIBC of 2.06, normal CREA of 42.6 umol/l, and normal RDWSD of 40.1 mg/dl were the parameters found using the model for the inhibition of DKD development. The lower risk was somewhat countered by a 12.8 percent HbA1C and a 22.9 percent PLCR.

## Discussion

T2DKD has been recognized as the major cause of end-stage renal failure (4). Its diagnosis is largely dependent on kidney biopsy, which is generally used to distinguish diabetic kidney disease from other kidney diseases. However, biopsy cannot be employed for early screening and diagnosis of T2DKD, thus resulting in missed diagnosis and misdiagnosis. The development of chronic albuminuria followed by a steady drop in GFR (classical phenotype of DKD) (24) has been adopted to diagnose DKD. Several studies have indicated the trajectories of renal function (i.e., changes in GFR and albuminuria with time) that diverge from this traditional phenotype over the past decade (30). Three non-classical DKD phenotypes have been identified, each of which are defined by albuminuria regression, fast GFR decrease, or the lack of proteinuria or albuminuria, respectively (31). Albuminuria has limitations in the prediction of the progression of DKD. The determination of albuminuria values is affected by a wide variety of factors (e.g., fever, strenuous exercise within 24h, menstruation, hyperglycemia and hypertension). For atypical DKD, albuminuria is not sufficiently specific for the diagnosis of DKD, and some studies have indicated that 30% of patients with albuminuria had negative urine albumin within 10 years, and this phenomenon has been more significant in type 2 diabetes patients (32, 33). Urinary albumin excretion was influenced by many factors (34). It was recommended that the diagnosis of albuminuria requires three 24-h urine collections over a 3-month period, with at least two of the three results exceeding the threshold and not measured by urinary routine. Thus, the diagnosis of albuminuria as a basis for DKD should be based on a combination of multiple tests and long-term follow-up with glomerular filtration rate, and the cause of albuminuria should be excluded. Thus, the necessity of a simple and convenient clinical tool to assess DKD in daily clinical practice is highlighted.

Accordingly, a multi-center retrospective study was conducted to analyze clinical indicators of T2DM and T2DKD based on real-world data, and adopted machine learning algorithms to investigate potential clinical and Laboratory risk factors for DKD among patients with T2DM. In this study, 15 ML models for ancillary diagnosis of T2DKD were initially developed in accordance with the clinical data from seven hospitals in China, and the efficacy of the 15 ML models was assessed. Meanwhile, we tried the CatBoost algorithms, which are seldom employed in medical studies. Our retrospective study showed that CatBoost is very effective for ancillary diagnosis of DKD. The patients’ clinical and laboratory parameters were assessed with a CatBoost model, and key features correlated with an increased risk of DKD, (e.g., SBP, CREA, LOS, TT, Age, PT, PLCR, ALB, GLU, FIBC, RDWSD, as well as HbA1c) were identified.

In this study, the differential diagnosis model of T2DKD was built based on 15 machine learning algorithms, thus solving the nonlinear relationship between clinical features and diagnosis results. The CatBoost model with the highest diagnostic accuracy than the other 14 models, such as light gradient booting model, Extreme Gradient Boosting and so on, indicating a good predictive performance. With LASSO analysis, SBP, CREA, LOS, TT, Age, PT, PLCR, ALB, GLU, FIBC, RDWSD, HbA1c were the top 12 major influencing factors of the index importance, which achieved statistical significance in multivariate logistic regression analysis.

Existing studies suggested that SBP, CREA, Age, ALB, and GLU are factors for DKD. High SBP was reported with rapidly eGFR decline in the Atherosclerosis Risk in Communities (ARIC) study (35). As reported by Gross JL et al., hypertension increased the morbidity of patients hospitalized with kidney disease, and increased blood pressure was found as a major risk factor for DKD (36). Sasso FC et al. found in their study that arterial pressure is a relevant factor for the progression of DKD in patients with DM, accompanied by hypertension is highly susceptible to periglomerular microvascular changes leading to development of DKD (37). Viazzi F et al. investigated the clinical records of more than 30,000 patients with T2DM combined with hypertension over 4 years of follow-up. It was found that elevated long-term blood pressure variability predicted CKD in T2DM and (38). In the model built in this study, SBP was the primary predictor of DKD, consistent with previous studies mentioned above. As revealed by the analysis of the examination of renal function in patients with DKD hospitalized between 2015 and 2017, CREA achieved a high predictive value in the diagnosis of patients with DKD and could effectively assess the status of renal function in patients with DM (39). This is consistent with the findings of our study.

Radcliffe NJ et al. found a correlation between elevated age, early GFR decline and DKD progression, consistent with the results of the present study (40). Elley et al. demonstrated an independent relationship between higher age and increased risk of DKD progression (28). López-Revuelta K et al. also suggested that age could be a risk factor for DKD development, with a mean age of 58.3 years in terms of DKD (41). The above studies assessed changes in GFR in predominantly adult patients (28). Several cross-sectional studies have shown changes in P-LCR, PLT, and FIBC in DKD patients in comparison with normal, suggesting that the occurrence of DKD is closely related to abnormal platelet function (42–44).

The study by Zoppini G et al. followed more than 1,000 patients with DKD and found that HbA1c was a risk factor for the progression of DKD. A decrease in HbA1c significantly reduced the risk of complications in patients with DM. A decrease in Hb A1c from 10% to 9% was also found to have a greater impact on reducing the risk of complications than a decrease in Hb A1c from 7% to 6% (45). Yun KJ, et al. found HbA1c variability may affect the development and progression of DKD in their study (46). Visit-to-visit variability of HbA1c was an independent risk factor of microalbuminuria in association with oxidative stress among type 2 diabetes mellitus patients (47, 48). Meanwhile, observational studies have not consistently demonstrated a glucose threshold (49). In a referred population of established DKD, higher HbA1c was not associated with higher risk of ESKD or death (50). In addition, our study found that HbA1c also influences the progression of DKD, in agreement with most previous studies.In addition, our study found that LOS, TT, PT, RDWSD also influence of DKD progression, which has not been reported in the literature and deserves further study.

Previously, it was confirmed that metabolic syndrome(MetS) and associated components (abdominal obesity, elevated BG, elevated BP and lipid metabolic disorder) are strongly related to CKD and a decreased estimated glomerular filtration rate (eGFR) (51–55). Over the 4-year follow-up period, Peijia L et al. found that MetS recovery was associated with a reduced risk of rapid eGFR decline in middle-aged and older adults, while MetS occurrence was not related to rapid eGFR decline. Recovery from MetS appeared to protect against a rapid decline in eGFR (56).

Due to the strong interpretability, logistic regression model is widely used to explore the risk factors of diseases. However, problems such as under-fitting, data missing, poor overall performance of the model are likely to occur in the process of modeling. In terms of the machine learning algorithms, this study has been considered the first to assess the risk of patients with DKD using the CatBoost model. As revealed by this study, the CatBoost model achieved great performance in the prediction of DKD. By analyzing clinical indicators of 1768 cases of type 2 diabetes and 1856 cases of type 2 diabetic kidney disease, this study applied the CatBoost model to the risk assessment of type 2 diabetic kidney disease for the first time, and analyzed the weight relationship of influencing factors. A good classification results was obtained (AUC=0.840).

This study had several limitations. First, although general clinical data and laboratory indexes were collected more comprehensively, some of the indexes were not covered in the model due to the missing values of ≥25%, and the significance of the correlation with type 2 diabetic kidney disease should be investigated in more detail when the volume of data is expanded.However, it was found through our data that some clinical parameters (cystatin-C, total 24-hour urine protein, duration of disease, etc.) are missing in many patients and many indicators cannot be generalized in primary care. Second, We found in the construction of the model that SBP was included as an important parameter in the prediction model, considering hypertension as an important confounding factor that is best analyzed in a stratified manner. Third, Due to data limitations, we were unable to select patients with a first diagnosis of T2DKD to model.Fourth, a cross-sectional study was conducted, and the results should be validated through a prospective study.

## Conclusions

To sum up, this retrospective study suggested that CatBoost could be highly effective in the early ancillary diagnosis of DKD. The importance of the model’s correlation to type 2 diabetic kidney disease should be investigated in depth after the data volume is expanded. In subsequent research, a greater amount of data and more machine learning models will be adopted for modeling research, as an attempt to build a better risk assessment model.

## Data availability statement

The raw data supporting the conclusions of this article will be made available by the authors, without undue reservation.

## Ethics statement

The studies involving human participants were reviewed and approved by the Ethics Committee of the Chongqing Medical University. Written informed consent for participation was not required for this study in accordance with the national legislation and the institutional requirements.

## Author contributions

XL, TZ, MD and HW conceived and designed the study. All authors contributed to the acquisition of data or analysis and interpretation of data. XL drafted the manuscript. MD drew the figures and tables.HH, YZ, TX, WN, and BZ revised the manuscript critically for essential intellectual content. All authors read and approved the final version to be published.
