# Supplementary material for: Predicting diabetic kidney disease for type 2 diabetes mellitus by machine learning in the real world: a multicenter retrospective study
Source: Front Endocrinol (Lausanne). 2023 Jul 4;14:1184190. doi: 10.3389/fendo.2023.1184190 (PMC10352831; doi:10.3389/fendo.2023.1184190)
Supplement: Supplementary file 1 [file Table_1.doc]

Table S1 Baseline demographic and laboratory characterics between T2DKD and DKD of the study population

| Variables | ALL | T2DKD | DKD | P-value |
| --- | --- | --- | --- | --- |
| N | 3624 | 1768 | 1856 |  |
| Gender,n(%) |  |  |  | 0.011 |
| Male | 2034 (56.1%) | 954 (54.0%) | 1080 (58.2%) |  |
| Female | 1590 (43.9%) | 814 (46.0%) | 776 (41.8%) |  |
| Smoke,n(%) |  |  |  | 0.213 |
| No | 2415 (66.6%) | 1160 (65.6%) | 1255 (67.6%) |  |
| Yes | 1209 (33.4%) | 608 (34.4%) | 601 (32.4%) |  |
| Drink,n(%) |  |  |  | 0.126 |
| No | 2638 (72.8%) | 1266 (71.6%) | 1372 (73.9%) |  |
| Yes | 986 (27.2%) | 502 (28.4%) | 484 (26.1%) |  |
| Age(Year)(median[IQR]) | 59.0 [50.0,68.0] | 56.0 [48.0,65.0] | 63.0 [54.0,71.0] | <0.001 |
| CREA(umol/L)(median[IQR]) | 58.6 [43.0,76.9] | 54.4 [41.0,68.0] | 65.1 [45.3,95.3] | <0.001 |
| SBP(mmHg)(median[IQR]) | 130 [120,144] | 126 [117,136] | 137 [124,152] | <0.001 |
| DBP(mmHg)(median[IQR]) | 80.0 [73.0,88.0] | 80.0 [73.0,86.0] | 81.0 [74.0,90.0] | <0.001 |
| LOS(day)(median[IQR]) | 7.00 [5.00,10.0] | 7.00 [5.00,9.00] | 8.00 [6.00,11.0] | <0.001 |
| GGT(u/l)(median[IQR]) | 27.0 [18.0,47.0] | 28.0 [18.0,50.0] | 26.0 [17.5,44.1] | 0.013 |
| ALT(u/l)(median[IQR]) | 21.0 [14.7,34.0] | 22.0 [15.0,36.0] | 20.0 [14.0,31.1] | <0.001 |
| Neup(%)(median[IQR]) | 63.9 [57.2,71.5] | 62.1 [55.6,69.0] | 65.8 [58.8,73.6] | <0.001 |
| Neu(×10^9^/l)(median[IQR]) | 3.91 [2.98,5.22] | 3.73 [2.91,4.90] | 4.11 [3.07,5.54] | <0.001 |
| LDLC(mmol/l)(median[IQR]) | 2.56 [1.94,3.16] | 2.60 [2.02,3.18] | 2.53 [1.87,3.14] | 0.004 |
| PT(s)(median[IQR]) | 12.1 [11.0,13.0] | 12.3 [11.3,13.0] | 11.8 [10.8,12.9] | <0.001 |
| TT(s)(median[IQR]) | 17.1 [15.5,18.3] | 17.4 [16.1,18.4] | 16.8 [14.9,18.2] | <0.001 |
| Monp(×10^9^/l)(median[IQR]) | 5.40 [4.10,6.80] | 5.40 [4.10,6.80] | 5.40 [4.20,6.70] | 0.720 |
| Mon(%)(median[IQR]) | 0.33 [0.24,0.44] | 0.33 [0.24,0.44] | 0.33 [0.23,0.46] | 0.826 |
| Bas(×10^9^/l)(median[IQR]) | 0.02 [0.01,0.03] | 0.02 [0.01,0.03] | 0.02 [0.01,0.03] | <0.001 |
| Basp(%)(median[IQR]) | 0.30 [0.10,0.40] | 0.24 [0.10,0.40] | 0.30 [0.10,0.50] | 0.003 |
| Eos(×10^9^/l)(median[IQR]) | 0.11 [0.06,0.19] | 0.10 [0.06,0.17] | 0.11 [0.06,0.20] | 0.144 |
| Eosp(%)(median[IQR]) | 1.80 [1.00,3.00] | 1.80 [1.00,2.81] | 1.80 [0.90,3.10] | 0.603 |
| PLCR(%)(median[IQR]) | 35.5 [28.6,42.8] | 37.1 [30.4,44.1] | 34.1 [27.4,41.1] | <0.001 |
| AST(u/l)(median[IQR]) | 20.0 [16.0,27.0] | 20.0 [16.0,28.0] | 20.0 [15.2,26.4] | 0.005 |
| UREA(umol/l)(median[IQR]) | 5.74 [4.64,7.26] | 5.41 [4.42,6.78] | 6.06 [4.87,7.90] | <0.001 |
| UA(umol/l)(median[IQR]) | 320 [257,392] | 303 [245,370] | 336 [267,413] | <0.001 |
| MCV(fl)(median[IQR]) | 89.8 [86.6,93.1] | 89.7 [86.8,92.8] | 90.0 [86.5,93.6] | 0.197 |
| MCHC(g/l)(median[IQR]) | 335 [327,343] | 336 [328,344] | 334 [326,342] | <0.001 |
| MCH(pg)(median[IQR]) | 30.2 [29.1,31.3] | 30.3 [29.2,31.2] | 30.2 [28.9,31.3] | 0.207 |
| TC(mmol/l)(median[IQR]) | 4.62 [3.90,5.44] | 4.61 [3.96,5.39] | 4.63 [3.84,5.47] | 0.956 |
| TP(g/l)(median[IQR]) | 69.5 [64.7,74.3] | 70.1 [65.7,74.5] | 68.7 [63.8,74.1] | <0.001 |
| APTT(s)(median[IQR]) | 31.6 [27.3,35.3] | 32.2 [27.8,35.8] | 31.0 [27.0,34.5] | <0.001 |
| Lymp(%)(median[IQR]) | 27.6 [20.5,33.9] | 29.6 [23.1,35.3] | 25.5 [18.6,32.3] | <0.001 |
| Lym(×10^9^/l)(median[IQR]) | 1.65 [1.24,2.11] | 1.76 [1.37,2.20] | 1.55 [1.14,2.02] | <0.001 |
| TG(mmol/l)(median[IQR]) | 1.70 [1.15,2.70] | 1.70 [1.14,2.72] | 1.70 [1.17,2.68] | 0.782 |
| WBC(×10^9^/l)(median[IQR]) | 6.24 [5.03,7.75] | 6.14 [5.01,7.50] | 6.38 [5.05,8.00] | 0.003 |
| ALB(g/l)(median[IQR]) | 41.5 [38.3,44.3] | 42.2 [39.6,44.7] | 40.7 [36.9,43.8] | <0.001 |
| DBIL(umol/l)(median[IQR]) | 3.30 [2.30,4.60] | 3.40 [2.40,4.70] | 3.20 [2.20,4.45] | <0.001 |
| ALP(u/l)(median[IQR]) | 77.0 [63.0,96.1] | 76.0 [63.6,94.2] | 77.2 [62.1,97.4] | 0.704 |
| P(mmol/l)(median[IQR]) | 1.15 [1.02,1.30] | 1.16 [1.04,1.30] | 1.15 [1.00,1.30] | 0.011 |
| GLU(mmol/l)(median[IQR]) | 9.59 [6.81,14.7] | 9.09 [6.67,14.2] | 10.2 [6.97,15.1] | <0.001 |
| HbA1c(%)(median[IQR]) | 8.70 [7.10,10.8] | 8.60 [6.90,10.5] | 8.80 [7.23,11.0] | <0.001 |
| RDW.SD(fl)(median[IQR]) | 41.9 [39.7,44.4] | 41.5 [39.4,43.7] | 42.4 [39.9,45.2] | <0.001 |
| RDW.CV(%)(median[IQR]) | 13.0 [12.4,13.6] | 12.8 [12.3,13.4] | 13.1 [12.5,13.7] | <0.001 |
| HCT(L)(median[IQR]) | 0.41 [0.37,0.44] | 0.42 [0.38,0.45] | 0.40 [0.36,0.44] | <0.001 |
| RBC(×10^12^/l)(median[IQR]) | 4.54 [4.13,4.94] | 4.62 [4.25,4.98] | 4.46 [3.96,4.90] | <0.001 |
| FIBC(g/l)(median[IQR]) | 3.03 [2.55,3.65] | 2.90 [2.42,3.40] | 3.20 [2.69,3.88] | <0.001 |
| PLT(×10^9^/l)(median[IQR]) | 183 [146,224] | 187 [149,225] | 181 [141,223] | 0.018 |
| PDW(%)(median[IQR]) | 16.1 [13.7,16.8] | 15.9 [13.5,16.9] | 16.2 [14.0,16.7] | 0.356 |
| MPV(fl)(median[IQR]) | 11.2 [10.3,12.2] | 11.5 [10.6,12.4] | 11.0 [10.1,12.0] | <0.001 |
| ApoA1(g/l)(median[IQR]) | 1.33 [1.13,1.56] | 1.37 [1.19,1.58] | 1.29 [1.08,1.54] | <0.001 |
| ApoB(g/l)(median[IQR]) | 0.96 [0.78,1.16] | 0.98 [0.81,1.18] | 0.94 [0.74,1.14] | <0.001 |
| IBIL(umol/l)(median[IQR]) | 6.84 [4.80,9.37] | 7.05 [5.00,9.60] | 6.60 [4.50,9.10] | <0.001 |
| HDLC(mmol/l)(median[IQR]) | 1.11 [0.93,1.36] | 1.11 [0.94,1.36] | 1.11 [0.92,1.36] | 0.363 |

Systolic blood pressure(SBP), diastolic blood pressure(DBP), length of hospitalization(LOS), white blood cell count (WBC), neutrophils percentage (Neu%), neutrophil count (Neu), monocyte percentage (Mon%), monocyte count (Mon), basophils percentage (Bas%), basophil count (Bas), eosinophils percentage (Eos%), eosinophil count (Eos), lymphocyte percentage (Lym%), lymphocyte count (Lym), platelet distribution width (PDW), mean platelet volume (MPV),platelet large cell ratio (P-LCR), platelet count (PLT), red blood cell distribution width-standard deviation (RDW-SD), red blood cell distribution width-coefficient of variation (RDW-CV), red blood cell count (RBC), hematocrit (HCT), mean corpuscular volume (MCV), mean corpuscular hemoglobin concentration (MCHC), mean corpuscular hemoglobin (MCH), prothrombin time (PT), thrombin time (TT), activated partial thromboplastin time (APTT), total protein (TP), albumin (ALB), gamma glutamyltransferase (GGT), alanine aminotransferase (ALT), aspartate aminotransferase (AST), urea, uric acid (UA), direct bilirubin (DBIL), alkaline phosphatase (ALP), phosphorus (P), Hemoglobin A1C(HbA1C), fibrinogen (FIB-C), creatinine (CREA), glucose (GLU), apolipoprotein A1 (ApoA1), apolipoprotein B (ApoB), indirect bilirubin (IBIL), total cholesterol (TC), triglyceride (TG), high-density lipoprotein cholesterol (HDL-C). low-density lipoprotein cholesterol (LDL-C),
